# Supplementary material for: Pathogen-specific innate immune response patterns are distinctly affected by genetic diversity
Source: Nat Commun. 2023 Jun 5;14:3239. doi: 10.1038/s41467-023-38994-5 (PMC10241821; doi:10.1038/s41467-023-38994-5)
Supplement: Supplementary file 1 — Supplementary Information [file 41467_2023_38994_MOESM1_ESM.pdf]

Supplementary Information

Pathogen-specific innate immune response patterns are distinctly affected by genetic diversity

Author list

Antje Häder[1,†], Sascha Schäuble[2,†], Jan Gehlen[3], Nadja Thielemann[4], Benedikt C. Buerfent[3,5], Vitalia Schüller[5], Timo Hess[3,5], Thomas Wolf[6], Julia Schröder[5], Michael Weber[1,6,7], Kerstin Hünniger[1,4], Jürgen Löffler[8], Slavena Vylkova[9], Gianni Panagiotou[2,10,11], Johannes Schumacher[3,5,‡,*], Oliver Kurzai[1,4, ‡,*]

Affiliations

[1] Research Group Fungal Septomics, Leibniz Institute for Natural Product Research and Infection Biology-Hans Knoell Institute; 07745 Jena, Germany.
[2] Department of Microbiome Dynamics, Leibniz Institute for Natural Product Research and Infection Biology-Hans Knoell Institute; 07745 Jena, Germany.
[3] Institute of Human Genetics, Philipps University of Marburg; 35033 Marburg, Germany.
[4] Institute for Hygiene and Microbiology, Julius Maximilians University of Wuerzburg; 97080 Wuerzburg, Germany.
[5] Institute of Human Genetics, University of Bonn, School of Medicine & University Hospital Bonn; 53127 Bonn, Germany.
[6] Systems Biology and Bioinformatics Unit, Leibniz Institute for Natural Product Research and Infection Biology-Hans Knoell Institute; 07745 Jena, Germany.
[7] Institute of Molecular Pathogenesis, Friedrich-Loeffler-Institute; 07743 Jena, Germany.
[8] Department of Internal Medicine II, University Hospital Wuerzburg; Josef-Schneider-Strasse 2 /C11; 97080 Wuerzburg, Germany.
[9] Research Group Host Fungal Interfaces, Septomics Research Center and Leibniz Institute for Natural Product Research and Infection Biology-Hans Knoell Institute; 07745 Jena, Germany.
[10] Faculty of Biological Sciences, Friedrich Schiller University; 07743 Jena, Germany.
[11] Department of Medicine and State Key Laboratory of Pharmaceutical Biotechnology, University of Hong Kong, Hong Kong SAR, China.

[†] These authors contributed equally
[‡] These authors jointly supervised this work

***** Corresponding authors. Email:
okurzai@hygiene.uni-wuerzburg.de;
johannes.schumacher@uni-marburg.de

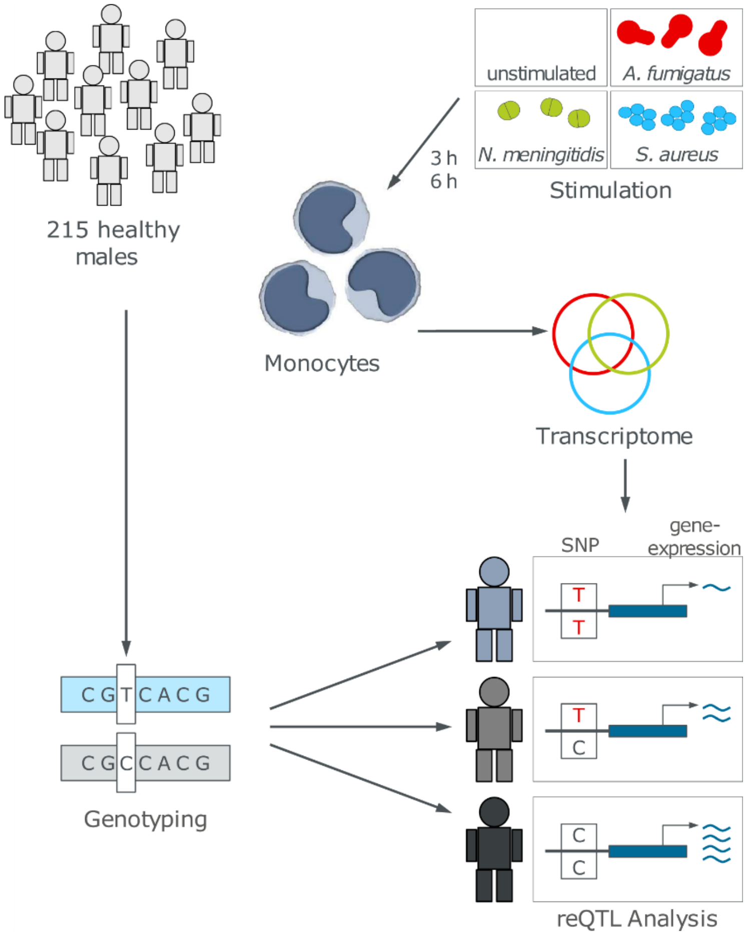

Supplementary Fig. 1 Study design. Monocytes from 215 healthy males were isolated from venous blood. Transcriptome-wide 3'-mRNAseq analyses were performed from unstimulated cells and after stimulation with three classes of infectious pathogens (fungus: *Aspergillus fumigatus*, Gram-negative bacterium: *Neisseria meningitidis*, Gram-positive bacterium: *Staphylococcus aureus*) for 3h and 6h. Genome-wide SNP-genotyping for each individual was carried out. reQTL analysis was used to determine the influence of host genetic variability on gene expression following pathogen exposure. SNP: single-nucleotide polymorphism, reQTL: response expression quantitative trait loci.

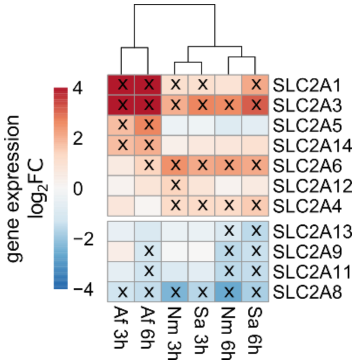

Supplementary Fig. 2 Expression of genes encoding glucose transporters. Gene expression $\log_2$(fold-changes) ($\log_2$FC) of genes encoding glucose transporters that are differentially expressed in at least one condition (marked with X: significance reported by 4 tools and $|\log_2 FC| \geq 1$, see Methods). Af: *A. fumigatus*, Nm: *N. meningitidis*, Sa: *S. aureus*. Source data are provided as a Source Data file.

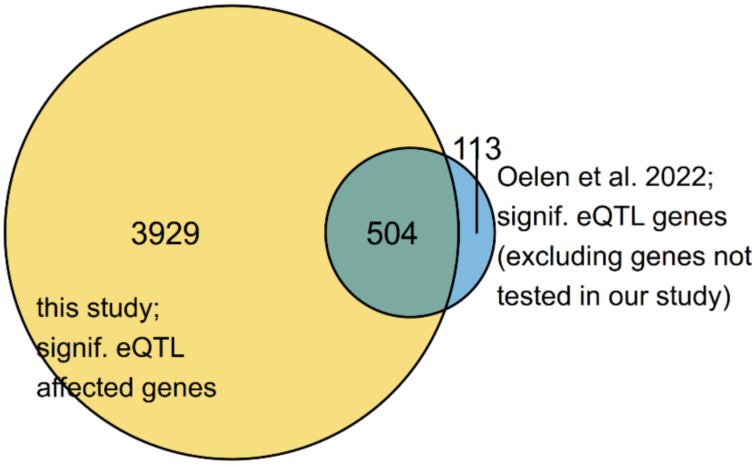

Supplementary Fig. 3 Comparison of *cis* eQTL data from this study and Oelen et al., 2022. Only genes tested for eQTL-regulation in our study were used for comparison of significantly eQTL-regulated genes of Oelen et al., 2022 (Supplementary Table S7 in Oelen et al.)[1]. While the majority of the identified eQTL-regulated genes in monocytes by Oelen et al. (504/617, 3h stimulated monocytes) were also detected in our study, we found 3,929 additional genes that were significantly regulated by eQTLs after 3h of stimulation. Significance of eQTLs from this study according to FDR-adjusted permutation test (see Methods). eQTL: expression quantitative trait loci. List of all eQTLs from this study is provided in Supplementary Data 2. Source data are provided as a Source Data file.

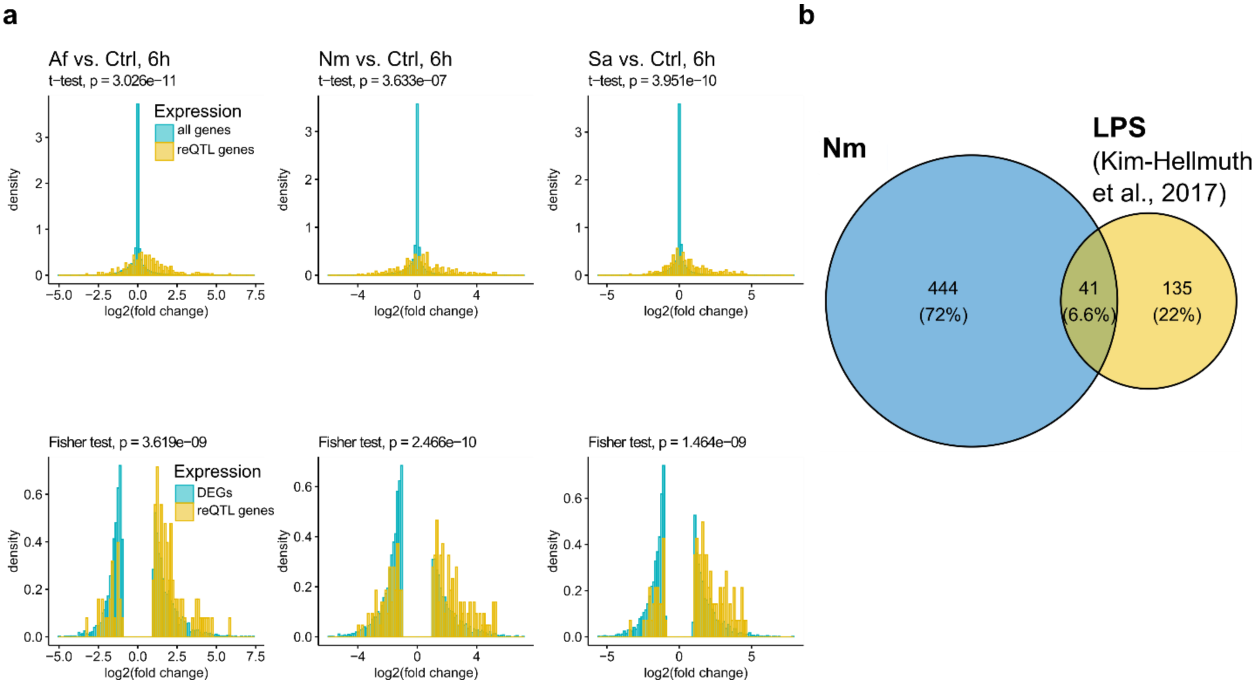

Supplementary Fig. 4 Overview of reQTL effects in pathogen-exposed monocytes. **(a)** Histograms comparing the gene expression $\log_2$FC distribution of all genes (top row) or all DEGs ($|\log_2 FC| \geq 1$, bottom row) *versus* all reQTL-regulated genes after 6h of stimulation. The distribution differences were tested for statistical significance. Two-tailed *t*-test or Fisher exact test based *p*-values are indicated. Ctrl: unstimulated, Af: *A. fumigatus*, Nm: *N. meningitidis*, Sa: *S. aureus*, reQTL: response expression quantitative trait loci, DEGs: differentially expressed genes, FC: fold-change. **(b)** Venn diagram shows the numbers of reQTL-regulated genes following *N. meningitidis* stimulation for 6h identified in this study compared to independent data published by Kim-Hellmuth et al., 2017 (Supplementary Data 2 in Kim-Hellmuth et al.)[2], where reQTLs were identified in human monocytes after stimulation with *E. coli*-LPS for 6h. Significance of reQTLs from this study according to Bonferroni corrected *p*-values from z-test of differences in regression coefficients to unstimulated control. LPS: lipopolysaccharide. Lists of all DEGs and reQTLs from this study are provided in Supplementary Data 1 and Supplementary Data 3, respectively. Source data are provided as a Source Data file.

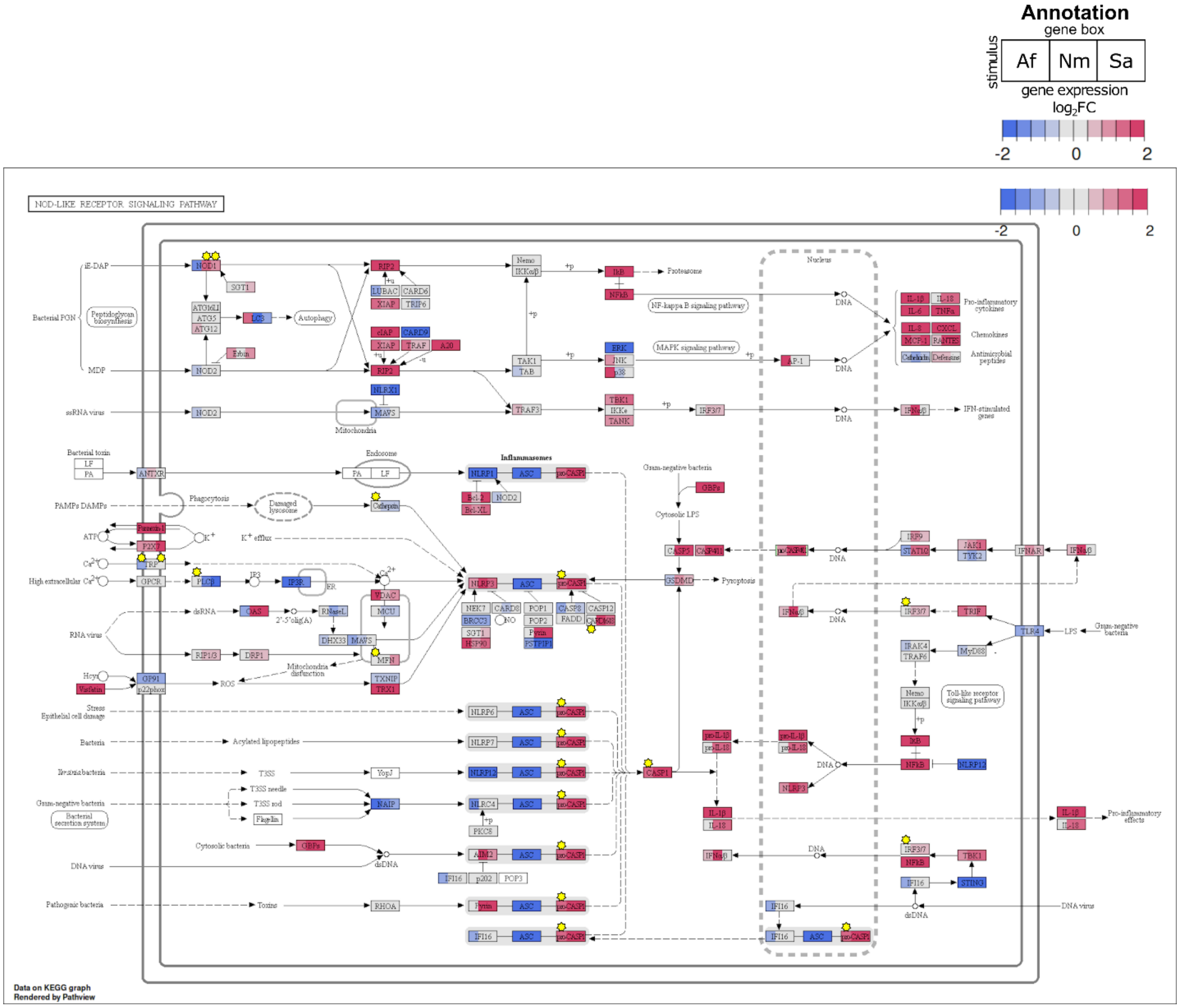

Supplementary Fig. 5 Pathview KEGG map for NOD-like receptor signaling pathway. The map summarizes the gene expression of monocytes after pathogen exposure and the conditions with identified reQTLs. Each box with a gene name contains three pieces of information. Color code from left to right: log_2FC following stimulation with *A. fumigatus* (Af), *N. meningitidis* (Nm) and *S. aureus* (Sa). Yellow stars highlight genes and conditions with identified reQTL effects. Data refer to the 6h timepoint. reQTL: response expression quantitative trait loci, FC: fold-change. Source data are provided as a Source Data file.

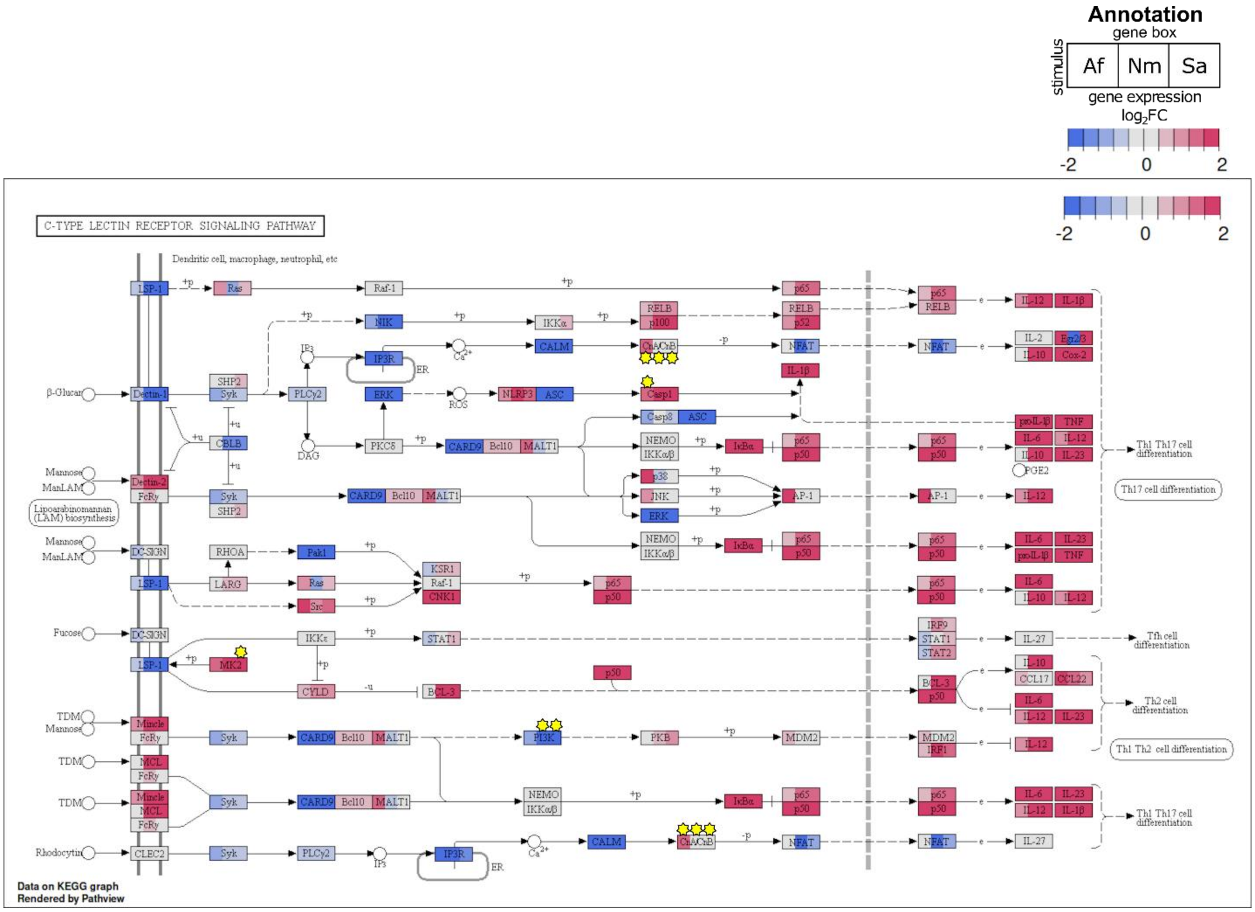

Supplementary Fig. 6 Pathview KEGG map for C-type lectin receptor signaling pathway. The map summarizes the gene expression of monocytes after pathogen exposure and the conditions with identified reQTLs. Each box with a gene name contains three pieces of information. Color code from left to right: log_2FC following stimulation with *A. fumigatus* (Af), *N. meningitidis* (Nm) and *S. aureus* (Sa). Yellow stars highlight genes and conditions with identified reQTL effects. Data refer to the 6h timepoint. reQTL: response expression quantitative trait loci, FC: fold-change. Source data are provided as a Source Data file.

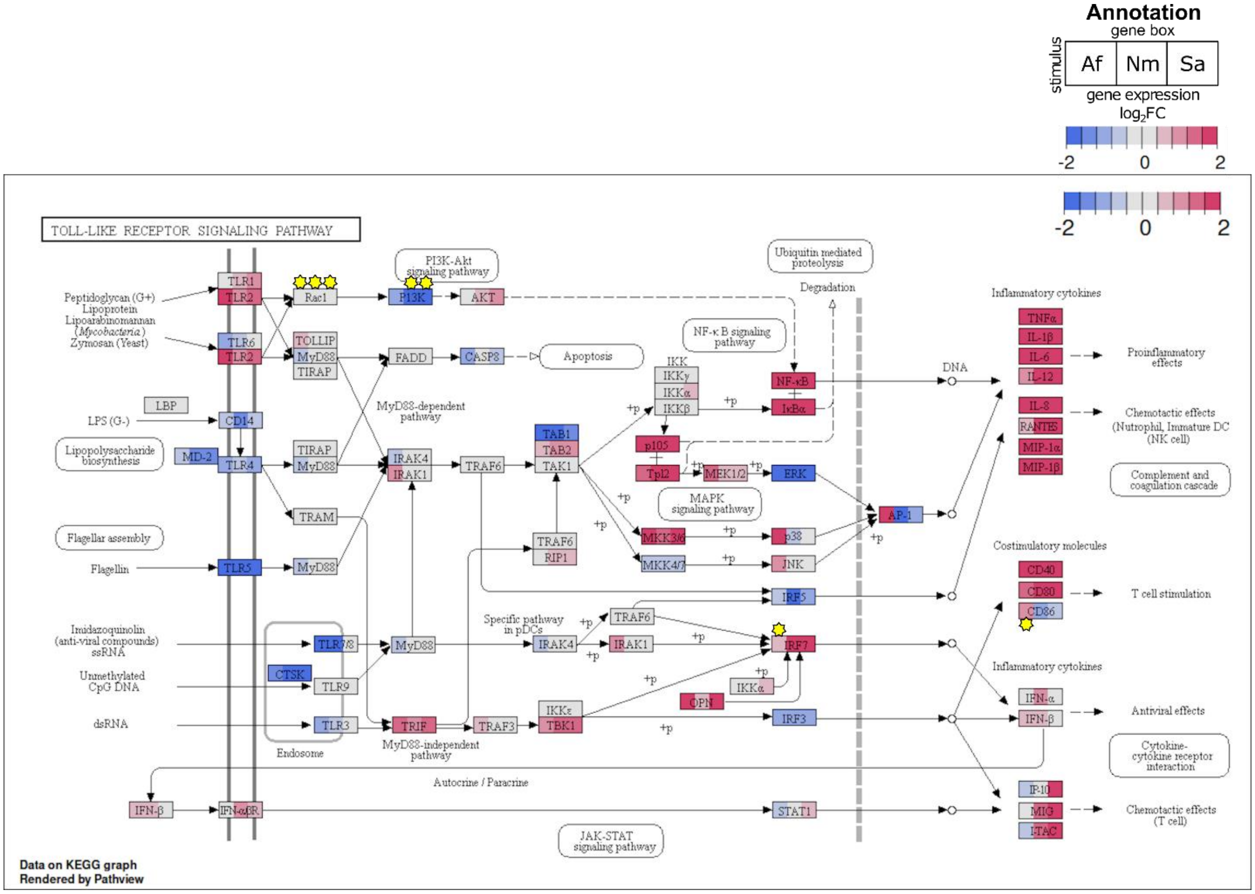

Supplementary Fig. 7 Pathview KEGG map for Toll-like receptor pathway. The map summarizes the gene expression of monocytes after pathogen exposure and the conditions with identified reQTLs. Each box with a gene name contains three pieces of information. Color code from left to right: $\log_2$FC following stimulation with *A. fumigatus* (Af), *N. meningitidis* (Nm) and *S. aureus* (Sa). Yellow stars highlight genes and conditions with identified reQTL effects. Data refer to the 6h timepoint. reQTL: response expression quantitative trait loci, FC: fold-change. Source data are provided as a Source Data file.

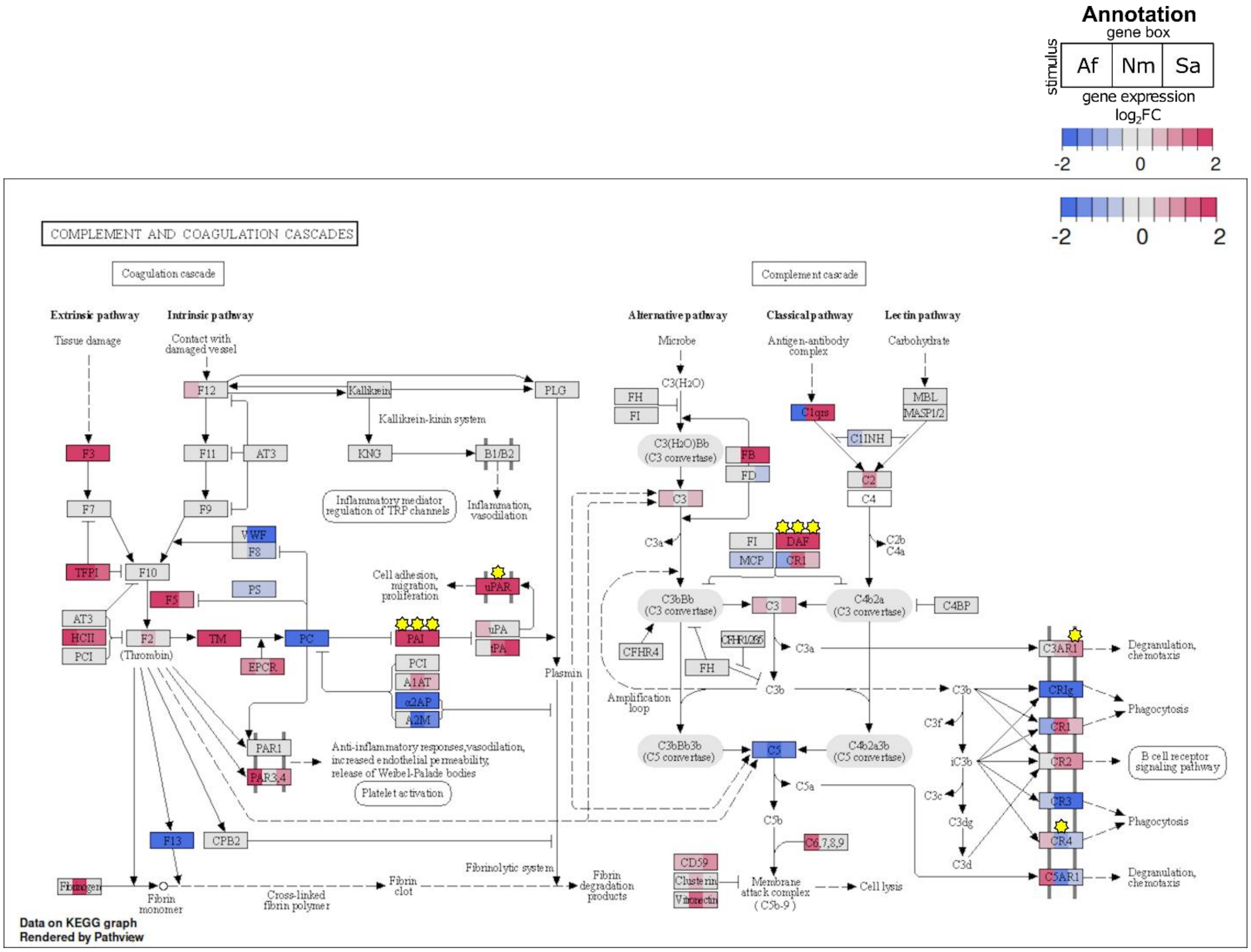

Supplementary Fig. 8 Pathview KEGG map for complement and coagulation cascades. The map summarizes the gene expression of monocytes after pathogen exposure and the conditions with identified reQTLs. Each box with a gene name contains three pieces of information. Color code from left to right: log_2FC following stimulation with *A. fumigatus* (Af), *N. meningitidis* (Nm) and *S. aureus* (Sa). Yellow stars highlight genes and conditions with identified reQTL effects. Data refer to the 6h timepoint. reQTL: response expression quantitative trait loci, FC: fold-change. Source data are provided as a Source Data file.

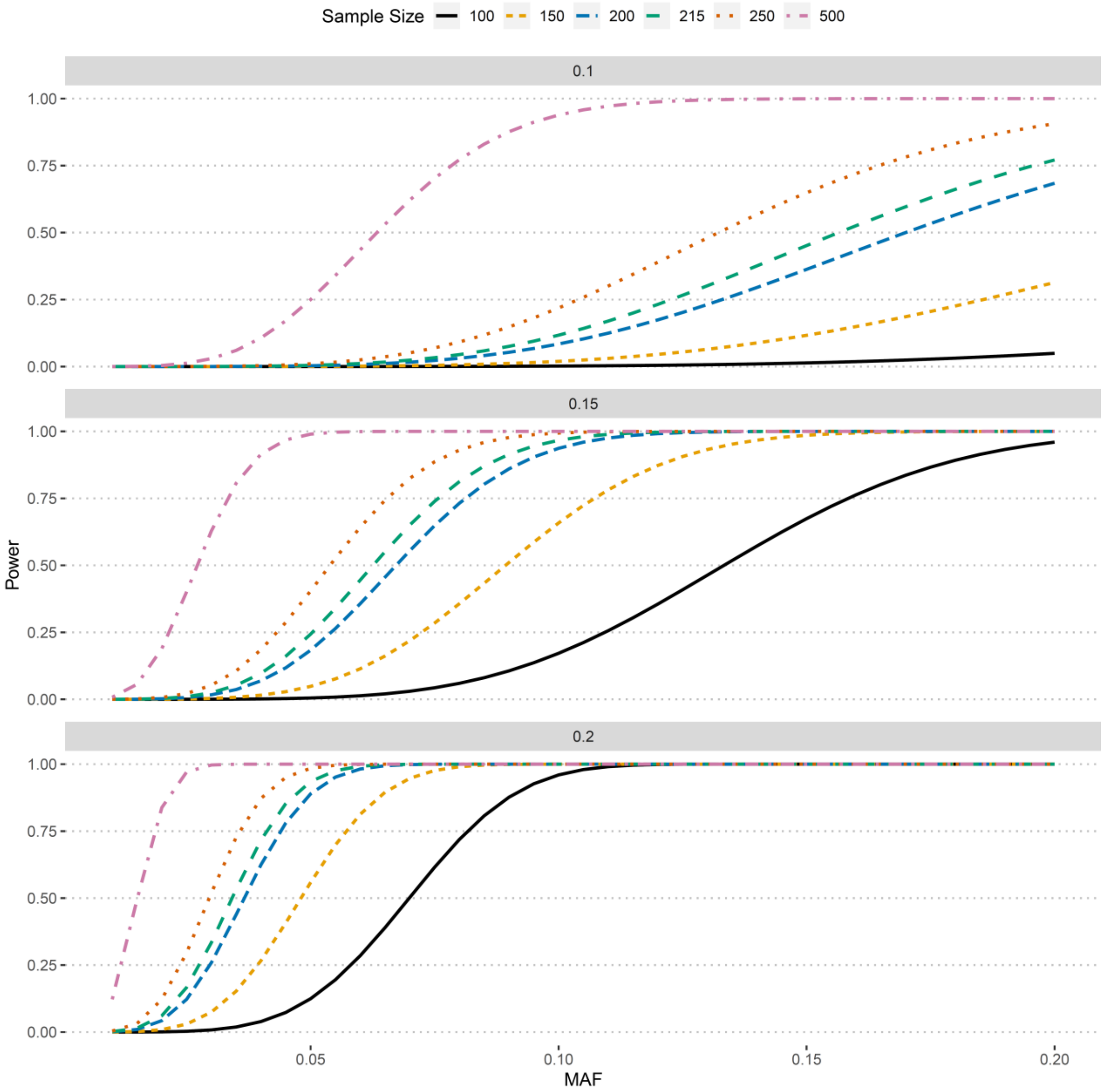

Supplementary Fig. 9 eQTL power analysis. The figure shows the estimated power to detect an eQTL effect on the y-axis against the minor allele frequency (MAF) on the x-axis. Different sample sizes are shown in different colors and pattern lines. The three panels show three different eQTL betas of 0.1, 0.15 and 0.2. The power analysis was performed with powerEQTL v0.3.4. Fixed parameters were set as follows: sigma.y = 0.13, nTests = 30,000,000. eQTL: expression quantitative trait loci. Source data are provided as a Source Data file.

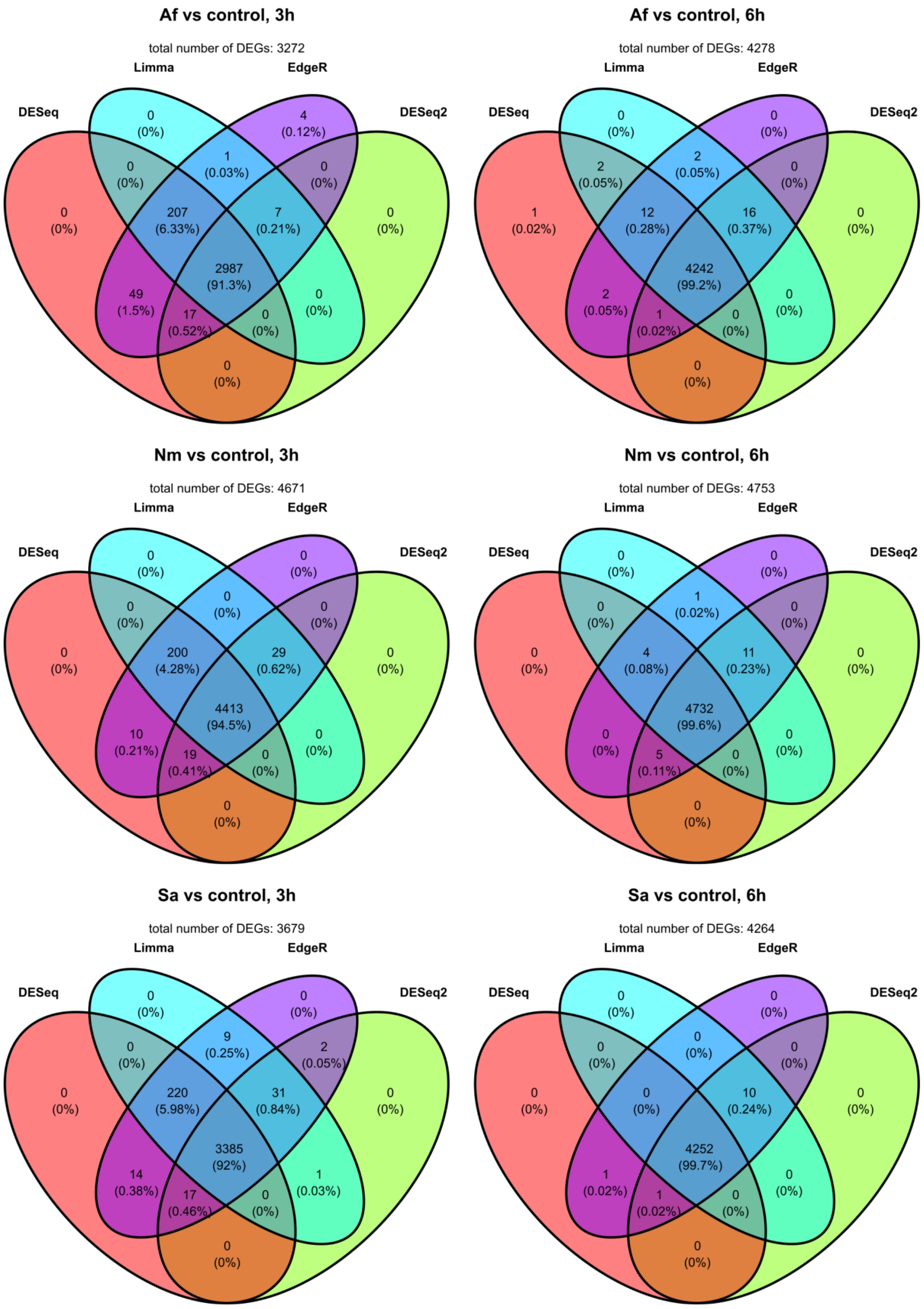

Supplementary Fig. 10 Combined use of four statistical tools for identification of differential gene expression. Venn diagrams show the number of reported statistically significant differentially expressed genes (DEGs). The numbers report statistically significant genes that also surpassed $|\log_2 FC| \geq 1$ reported either by one or several tools. FC: fold-change, Af: *A. fumigatus*, Nm: *N. meningitidis,* Sa: *S. aureus*. List of DEGs that are identified by four statistical tools (see Methods) to identify differential gene expression is provided in Supplementary Data 8. Source data are provided as a Source Data file.

Supplementary References

1 Oelen, R. *et al.* Single-cell RNA-sequencing of peripheral blood mononuclear cells reveals widespread, context-specific gene expression regulation upon pathogenic exposure. *Nat Commun* **13**, 3267, doi:10.1038/s41467-022-30893-5 (2022).

2 Kim-Hellmuth, S. *et al.* Genetic regulatory effects modified by immune activation contribute to autoimmune disease associations. *Nat Commun* **8**, 266, doi:10.1038/s41467-017-00366-1 (2017).
