# Supplementary material for: Pathogen-specific innate immune response patterns are distinctly affected by genetic diversity
Source: Nat Commun. 2023 Jun 5;14:3239. doi: 10.1038/s41467-023-38994-5 (PMC10241821; doi:10.1038/s41467-023-38994-5)
Supplement: Supplementary file 3 — Description of Additional Supplementary Files [file 41467_2023_38994_MOESM3_ESM.pdf]

Description of Additional Supplementary Files

Supplementary Data 1: List of all source DEGs and full list of enriched KEGG pathways (Excel spreadsheet).

Supplementary Data 2: List of all eQTLs (Excel spreadsheet).

Supplementary Data 3: List of all reQTLs (Excel spreadsheet).

Supplementary Data 4: Shared eGenes across pathogens at 3h and 6h with identical or different cis reQTLs (Excel spreadsheet).

Supplementary Data 5: List of all unique reQTLs (Excel spreadsheet).

Supplementary Data 6: List of disease-associated GWAS SNPs with reQTL effects in stimulated monocytes with all associations of reQTLs and GWAS diseases and disease abbreviations (Excel spreadsheet).

Supplementary Data 7: Results of colocalization analysis with coloc (Excel spreadsheet).

Supplementary Data 8: List of DEGs that are identified by four statistical tools to identify differential gene expression (Excel spreadsheet).
