# Supplementary material for: Pathogen-specific innate immune response patterns are distinctly affected by genetic diversity
Source: Nat Commun. 2023 Jun 5;14:3239. doi: 10.1038/s41467-023-38994-5 (PMC10241821; doi:10.1038/s41467-023-38994-5)
Supplement: Supplementary file 12 — Reporting Summary [file 41467_2023_38994_MOESM12_ESM.pdf]

nature portfolio

Corresponding author(s): Johannes Schumacher, Oliver Kurzai

Last updated by author(s): Apr 24, 2023

Reporting Summary

Nature Portfolio wishes to improve the reproducibility of the work that we publish. This form provides structure for consistency and transparency in reporting. For further information on Nature Portfolio policies, see our Editorial Policies and the Editorial Policy Checklist.

Statistics

For all statistical analyses, confirm that the following items are present in the figure legend, table legend, main text, or Methods section.

n/a	Confirmed	
☐	☒	The exact sample size (*n*) for each experimental group/condition, given as a discrete number and unit of measurement
☐	☒	A statement on whether measurements were taken from distinct samples or whether the same sample was measured repeatedly
☐	☒	The statistical test(s) used AND whether they are one- or two-sided<br>*Only common tests should be described solely by name; describe more complex techniques in the Methods section.*
☐	☒	A description of all covariates tested
☐	☒	A description of any assumptions or corrections, such as tests of normality and adjustment for multiple comparisons
☐	☒	A full description of the statistical parameters including central tendency (e.g. means) or other basic estimates (e.g. regression coefficient) AND variation (e.g. standard deviation) or associated estimates of uncertainty (e.g. confidence intervals)
☐	☒	For null hypothesis testing, the test statistic (e.g. *F*, *t*, *r*) with confidence intervals, effect sizes, degrees of freedom and *P* value noted<br>*Give P values as exact values whenever suitable.*
☒	☐	For Bayesian analysis, information on the choice of priors and Markov chain Monte Carlo settings
☐	☒	For hierarchical and complex designs, identification of the appropriate level for tests and full reporting of outcomes
☐	☒	Estimates of effect sizes (e.g. Cohen's *d*, Pearson's *r*), indicating how they were calculated

Our web collection on statistics for biologists contains articles on many of the points above.

Software and code

Policy information about availability of computer code

Data collection: No software was used for data collection.

Data analysis:
R programming language (v3.6.3)
powerEQTL (v0.3.4)
IMPUTE2 (v2.3.2)
PLINK (v1.9)
FastQC (v0.11.7)
BBMap (v37.44)
STAR Aligner (v2.5.2b)
FeatureCounts (v1.20.6)
multiQC (v1.6)
DESeq (v1.30.0)
DESeq2 (v1.18.1)
limma (v3.34.6)
edgeR (v3.20.7)
enrichR (v2.1.0)
pathview (v1.26.0)
biomaRt (v2.36.1)
QTLTools (v1.3.1)
qvalue (v2.12.0)
dr4pl (v2.0.0)

coloc (v5.1.1)
ApE (v3.0.8)
ggpubr (v0.4.0)

For manuscripts utilizing custom algorithms or software that are central to the research but not yet described in published literature, software must be made available to editors and reviewers. We strongly encourage code deposition in a community repository (e.g. GitHub). See the Nature Portfolio guidelines for submitting code & software for further information.

Data

Policy information about availability of data

All manuscripts must include a data availability statement. This statement should provide the following information, where applicable:

- Accession codes, unique identifiers, or web links for publicly available datasets
- A description of any restrictions on data availability
- For clinical datasets or third party data, please ensure that the statement adheres to our policy

Transcriptome and genotype data were collected in this study. The source data supporting the findings of this study including differential gene expression data, eQTL and reQTL information are available within the paper and its Supplementary Data files. RNA-sequencing data are accessible through GEO accession number GSE177040 (https://www.ncbi.nlm.nih.gov/geo/query/acc.cgi?acc=GSE177040). Data on GWAS was collected from NHGRI-EBI GWAS Catalog (https://www.ebi.ac.uk/gwas/). Any additional information required to reanalyze the data reported in this paper is available from the corresponding author upon request due to donor privacy protection.

Research involving human participants, their data, or biological material

Policy information about studies with human participants or human data. See also policy information about sex, gender (identity/presentation), and sexual orientation and race, ethnicity and racism.

Reporting on sex and gender	Findings apply to both sexes. Study design included male donors (n = 215). Selected findings were confirmed using cells isolated from an independent female cohort. (n = 9).
Reporting on race, ethnicity, or other socially relevant groupings	Volunteers of European ancestry were recruited for this study.
Population characteristics	Characteristics of included male participants were age between 18 to 40 years (mean 28), non-smokers, C-reactive-protein ≤ 10mg/l, without infection or vaccination for at least 4 weeks before blood withdrawal and blood count within normal range. For functional NOD1 and CD86 ex-vivo assays, venous blood was collected from nine healthy female volunteers aged between 20 to 40 years (mean 28) without infection or vaccination for at least 4 weeks before blood withdrawal. Written informed consent was obtained from all volunteers before blood withdrawal.
Recruitment	Participants were recruited on a voluntary basis in institutes and universities in Jena and Würzburg, Germany, with the help of flyers, e-mail notifications, information desks and presentations. Participants were included in the study if they met the inclusion criteria of the described population characteristics, which enabled us to exclude recruitment biases that otherwise may have impacted our results.
Ethics oversight	This study was approved by the Ethics Committees of the Friedrich-Schiller-University Jena (permit number: 3811-07/13) and the Julius-Maximilians-University of Würzburg (permit number: EK 191/21).

Note that full information on the approval of the study protocol must also be provided in the manuscript.

Field-specific reporting

Please select the one below that is the best fit for your research. If you are not sure, read the appropriate sections before making your selection.

☒ Life sciences ☐ Behavioural & social sciences ☐ Ecological, evolutionary & environmental sciences

For a reference copy of the document with all sections, see nature.com/documents/nr-reporting-summary-flat.pdf

Life sciences study design

All studies must disclose on these points even when the disclosure is negative.

Sample size	Since the aim of this project was to conduct transcriptome and eQTL analyses in conjunction, necessary sample size was determined based on the more challenging detection of eQTLs. For eQTL analysis, only cis-eQTL analysis was targeted requiring substantially lower sample size to reach statistical significance. We adapted our sample size from a power calculation and from sample sizes used in published eQTL studies (see Kim et al., 2014 [137 participants] and Nédelé et al., 2016 [175 participants], both identified >1,000 eQTLs). Power analysis for the identification of eQTL effects was based on the assumption of different minor allele frequency (MAF) and effect sizes. The power analysis was performed with powerEQTL (v0.3.4). Fixed parameters were set as follows: sigma.y = 0.13, nTests = 30,000,000.
Data exclusions	For each participant (n = 215), we analysed 8 different stimuli, resulting in 1720 samples for RNA-seq. Samples with very low RNA concentration were excluded from RNA-seq. For eQTL analysis, all samples with a D statistic outside of 1.5*IQR and less than three million

	mapped reads were excluded as done before (Wright et al., 2014).
Replication	To enable robustness of our study results we minimized confounding effects by establishing a homogenous study cohort (see 'Sample size'). Nevertheless a certain degree of heterogeneity was to be expected from human samples. Due to a high sample size requirement for eQTL analysis and consequently to enable maximized statistically significant output, all samples were used for differential gene expression and eQTL detection analysis.<br>Selected findings were successfully confirmed using cells isolated from an independent cohort of nine females.
Randomization	The study participants were allocated into experimental groups by their genotypes. By using a very homogenous cohort (males, 18-40 years, European ancestry, non-smoker) we did not include covariates.
Blinding	The names of all study participants were encrypted. All investigators that processed or analyzed the samples had no access to information about the study participants.

Reporting for specific materials, systems and methods

We require information from authors about some types of materials, experimental systems and methods used in many studies. Here, indicate whether each material, system or method listed is relevant to your study. If you are not sure if a list item applies to your research, read the appropriate section before selecting a response.

Materials & experimental systems

n/a	Involved in the study
☐	☒ Antibodies
☒	☐ Eukaryotic cell lines
☒	☐ Palaeontology and archaeology
☐	☒ Animals and other organisms
☒	☐ Clinical data
☒	☐ Dual use research of concern
☒	☐ Plants

Methods

n/a	Involved in the study
☒	☐ ChIP-seq
☒	☐ Flow cytometry
☒	☐ MRI-based neuroimaging

Antibodies

Antibodies used	CD14 monoclonal anti-human antibody conjugated to Microbeads (Miltenyi Biotec, Cat.-Nr.: 130-050-201, RRID:AB_2665482) were used to isolate CD14+ monocytes from peripheral blood mononuclear cells (PBMCs) according to the manufacturer's instructions. CD14-antibody [47-3D6] (PerCP) (Abcam, Cat.-Nr.: ab91146, RRID: AB_10675401) was used to validate the purity of the monocyte population.
Validation	The purity of the monocyte population was verified with CD14-antibody by flow cytometry.

Animals and other research organisms

Policy information about studies involving animals; ARRIVE guidelines recommended for reporting animal research, and Sex and Gender in Research

Laboratory animals	Aspergillus fumigatus (ATCC46645)<br>Staphylococcus aureus (ATCC25923)<br>Neisseria meningitidis, serogroup C (WUE2120)
Wild animals	The study did not involve wild animals.
Reporting on sex	Findings apply to both sexes. Study design included male donors (n = 215). Selected findings were confirmed using cells isolated from female donors (n = 9).
Field-collected samples	The study did not involve samples collected from the field.
Ethics oversight	This study was approved by the Ethics Committees of the Friedrich-Schiller-University Jena (permit number: 3811-07/13) and the Julius-Maximilians-University of Würzburg (permit number: EK 191/21).

Note that full information on the approval of the study protocol must also be provided in the manuscript.
